# Regional tissue perfusion index (RTPI): a new optical-based metric for quantifying regional tissue perfusion

**DOI:** 10.1007/s10877-026-01424-0

**Published:** 2026-02-24

**Authors:** Babak Shadgan, Iman Amani Tehrani, Sadra Khosravi, Zahra Askari, Amir Parham Pirhadi Rad, Ali Bashashati

**Affiliations:** 1 Department of Orthopaedics, The University of British Columbia, Vancouver, BC Canada; 2 School of Biomedical Engineering, The University of British Columbia, Vancouver, BC Canada; 3 Department of Pathology and Laboratory Medicine, The University of British Columbia, Vancouver, BC Canada; 4 Implantable Biosensing Laboratory, International Collaboration on Repair Discoveries, 5440 - ICORD, Blusson Spinal Cord Centre 818 W 10th Ave, Vancouver, BC V5Z1M9 Canada; 5 Department of Molecular Oncology, BC Cancer Agency, 675 West 10th Avenue, Vancouver, British Columbia V5Z 1L3 Canada

**Keywords:** Blood flow, Microvascular monitoring, Near-infrared spectroscopy, Principal component analysis, Tissue perfusion

## Abstract

Accurate, continuous assessment of regional tissue perfusion remains a significant clinical challenge, as most existing modalities are invasive, indirect, or impractical for routine monitoring. Near-infrared spectroscopy (NIRS) has been widely adopted to assess tissue oxygenation; however, conventional NIRS-derived indices are insufficient surrogates for true perfusion and often fail to capture rapid hemodynamic changes. This study aimed to introduce and validate the Regional Tissue Perfusion Index (RTPI), a novel NIRS-derived metric that integrates multiple features of the NIRS signal to provide continuous, non-invasive, and physiologically relevant assessment of tissue perfusion. RTPI was developed using principal component analysis (PCA) of multiple NIRS-derived parameters, including pulse amplitude ratio, derivative, and area under the curve. Its performance was evaluated in healthy volunteers during controlled ischemia–reperfusion protocols and compared with established reference standards, including laser Doppler flowmetry (LDF) and photoplethysmography (PPG). RTPI showed acceptable correlations with LDF Flux and PPG perfusion index (PI) during dynamic perfusion changes. Unlike conventional NIRS-derived oxygenation and hemodynamic indices, which often exhibited delayed responses, RTPI demonstrated immediate and significant sensitivity to both complete and partial ischemia–reperfusion episodes in the analyzed datasets. Intraclass correlation indicated stronger session-to-session reliability for RTPI than for both Flux and PI. RTPI represents a multiparametric, physiologically meaningful, and computationally efficient metric for real-time monitoring of tissue perfusion. Its ability to detect perfusion compromise independently of oxygenation indices highlights its translational potential for bedside implementation in critical care, trauma, perioperative, and vascular medicine, where improved diagnostic accuracy could significantly impact patient outcomes.

**Supplementary Information:**

The online version contains supplementary material available at 10.1007/s10877-026-01424-0.

## Introduction

Tissue perfusion, the delivery of blood through capillaries to biological tissues, is indispensable for supplying oxygen and nutrients and removing metabolic waste, thereby sustaining cellular metabolism and homeostasis [1]. Adequate perfusion is a critical determinant of tissue viability and organ function across all organ systems [2]. Conversely, perfusion deficits underlie a broad spectrum of disorders, including tissue ischemia and hypoxia, circulatory shock [3], ischemia-reperfusion injury [4], compartment syndrome [5], pressure ulcer formation [6], and impaired wound healing [7]. Hypoperfusion compromises cellular integrity and can escalate to multiple-organ dysfunction and systemic deterioration [8]. Accordingly, a reliable assessment of tissue perfusion is a central objective in critical care [9], postoperative monitoring [10], emergency medicine [11], vascular surgery [12], and rehabilitation [13].

Despite its clinical importance, accurate assessment and monitoring of tissue perfusion, especially at the microvascular level, remains a longstanding challenge in modern medicine. Current methods are often invasive, intermittent, or provide only indirect surrogates of perfusion. Techniques such as arterial catheterization, thermodilution, and radiolabeled tracers, although valuable in specific clinical settings, involve procedural risks and are not suitable for localized or continuous monitoring. Thermal-diffusion probes (e.g., Hemedex QFlow™) provide absolute, real-time, quantitative perfusion measurements directly within tissue, an invaluable tool in neuro-ICU care; however, a catheter must be implanted into the brain or soft tissue, limiting its use to operating rooms or ICUs [14,15]. Near-infrared spectroscopy with indocyanine green (NIRS-ICG) applies the Fick principle to yield site-specific blood-flow values; nevertheless, it still requires repeated intravenous dye injections, preventing truly continuous bedside use [16,17]. Collectively, these modalities are precise yet impractical for routine, multi-site, or prolonged monitoring because of their procedural burden.

Non-invasive techniques that avoid vascular access are safer and more widely available; however, each method sacrifices depth, stability, absolute quantification, and accurate regional perfusion measurement. Conventional systemic surrogates, such as standard hemodynamic measurements (e.g., heart rate, blood pressure, and blood oxygen saturation), are widespread; however, they fail to detect spatial or temporal variations in tissue blood flow. Furthermore, they are designed for systemic evaluation rather than for localized perfusion monitoring, which limits their utility for detecting subtle regional hemodynamic changes [18,19]. Laser Doppler flowmetry (LDF), a widely used optical technique for assessing microvascular perfusion, has provided valuable insights into blood flow in the skin and superficial tissues by detecting Doppler shifts caused by red blood cell motion. While LDF is highly sensitive to changes in microvascular perfusion, key limitations include sensitivity to tissue optical properties, susceptibility to motion artifacts, and the reporting of perfusion in relative (non-absolute) units, with the effective measurement depth and sampling volume varying with probe configuration and tissue properties [20]. Modeling studies show that, for commonly used wavelengths and probe geometries, effective measurement depth is typically on the order of sub-millimeters to about 1 mm, with corresponding measurement volumes that depend on wavelength and source–detector separation [21]. These constraints, together with spatial heterogeneity of the microvasculature and sensitivity to probe placement, complicate standardization across subjects and sites and reduce reproducibility when probes are removed and replaced [22]. Consequently, LDF is widely used in research and in selected specialist clinical applications (e.g., neurosurgery and neurointensive care) [23], while broader use as a general-purpose perfusion monitor remains constrained by standardization and interpretation challenges [20,22]. The photoplethysmography (PPG)-derived perfusion index (PI), defined as the ratio of the pulsatile (AC) to non-pulsatile (DC) components of the plethysmographic waveform (AC/DC × 100%), reflects peripheral vasomotor tone and is implemented in monitoring systems as an index of peripheral perfusion at the sensor site [24,25]. PI is readily obtainable at the bedside, but its values are influenced by physiological and measurement factors (e.g., local temperature and vasomotor tone) [24], and its distribution in healthy adults is highly skewed; in critically ill patients, changes in PI track changes in peripheral perfusion profiles [25]. Furthermore, PI varies across digits, with significant interfinger differences and poor-to-moderate agreement reported when PI is compared across fingers, which limits its reliability as a stand-alone perfusion surrogate without standardized site selection [26,27]. Diffuse correlation spectroscopy (DCS) provides a direct, noninvasive index of microvascular blood flow using near-infrared light, with deep-tissue penetration of ~ 1 cm and the potential for continuous monitoring [28]. In neurocritical care applications, DCS-derived cerebral flow metrics have shown significant correlations with invasive thermal diffusion flowmetry and have been evaluated alongside other invasive intracranial monitoring modalities [29,30]. However, conventional DCS implementations often require expensive single-photon-counting detectors and bulky fiber-optic probes, which have limited widespread commercial and clinical adoption [31]. Therefore, even the best non-invasive tools struggle with depth, motion, or practicality constraints, underscoring the need for a dye-free, motion-tolerant, continuous index of microvascular perfusion.

The ability to continuously and non-invasively monitor local tissue perfusion in a practical clinical setting remains an unmet need. Such a capability would allow earlier detection of tissue damage, help customize hemodynamic treatments, and enable real-time monitoring of therapeutic efficacy. It would also broaden perfusion monitoring to include outpatient, post-surgery, and rehabilitative settings, leading to better outcomes for a wide range of patients. This gap in clinical practice highlights the need to develop new physiologic perfusion indices that are accurate, reliable, and suitable for scalable, non-invasive platforms.

Near-Infrared Spectroscopy (NIRS) has emerged as a valuable non-invasive modality for assessing localized tissue oxygenation and hemodynamics. To date, the majority of NIRS applications have focused on parameters related to tissue oxygenation, including oxygenated hemoglobin (O₂Hb), deoxygenated hemoglobin (HHb), hemoglobin difference (Hb-diff), and tissue oxygen saturation (StO_2_) or the tissue oxygenation index (TOI). However, using these parameters, particularly TOI, as surrogates for tissue perfusion is not accurate. Although TOI is often interpreted as an indirect marker of perfusion, this assumption is flawed, as clinical scenarios exist in which changes in TOI do not correspond to actual alterations in tissue perfusion [32]. Similarly, some studies have proposed total hemoglobin (THb) as a surrogate for perfusion; however, this can be misleading. THb primarily reflects local blood volume, and its fluctuations indicate blood pooling or volume shifts rather than perfusion dynamics [33,34]. Currently, conventional NIRS methods lack a reliable and specific index for accurate measurement or monitoring of regional tissue perfusion [33,35].

To address this gap, we have developed a novel physiological metric, the Regional Tissue Perfusion Index (RTPI), derived from NIRS signals and designed to quantify real-time changes in tissue perfusion. Unlike oxygenation-based indices, RTPI directly reflects dynamic changes in blood flow, independent of tissue oxygenation indices. Our approach extracts physiologically meaningful features and combines them using unsupervised principal component analysis (PCA). Statistical analysis confirms both the clinical relevance and statistical significance of our results.

In this manuscript, we present the conceptual foundation, methodological framework, and validation results of the NIRS-derived RTPI measure. We evaluated the real-time performance of RTPI against established references, including LDF and PPG, under experimentally controlled ischemia–reperfusion conditions in healthy participants. Our findings highlight the clinical potential of RTPI as a novel, noise-resistant measure of tissue perfusion and outline future directions for its development and translational application in both research and medical practice. Ongoing and future studies will focus on incorporating advanced feature extraction techniques and more sophisticated modelling approaches to further enhance the accuracy and reliability of perfusion assessment.

## Methods

### Participants’ recruitment

Twenty healthy young adult volunteers (14 male, 6 female) were enrolled, with an average age of 26.90 ± 7.18 years and a BMI of 24.26 ± 2.96 kg/m² (mean ± SD). Participants were recruited from the local community and screened to exclude conditions or factors expected to affect peripheral perfusion or measurement quality, including major cardiovascular, respiratory, or neurological disease, skin lesions at sensor sites, pregnancy, use of vasoactive medications, allergies to sensor materials or adhesives, and any other condition compromising safety or data quality. Each participant completed two recording sessions, spaced one week apart, yielding a total of 40 datasets. This inter-session interval was selected to allow for full hemodynamic washout and to minimize potential carryover effects. All study procedures were approved by the institutional research ethics board and conducted in accordance with national guidelines for research involving human subjects. Written informed consent was obtained from all participants prior to data collection.

### Experimental setup

**Fig. 1:**
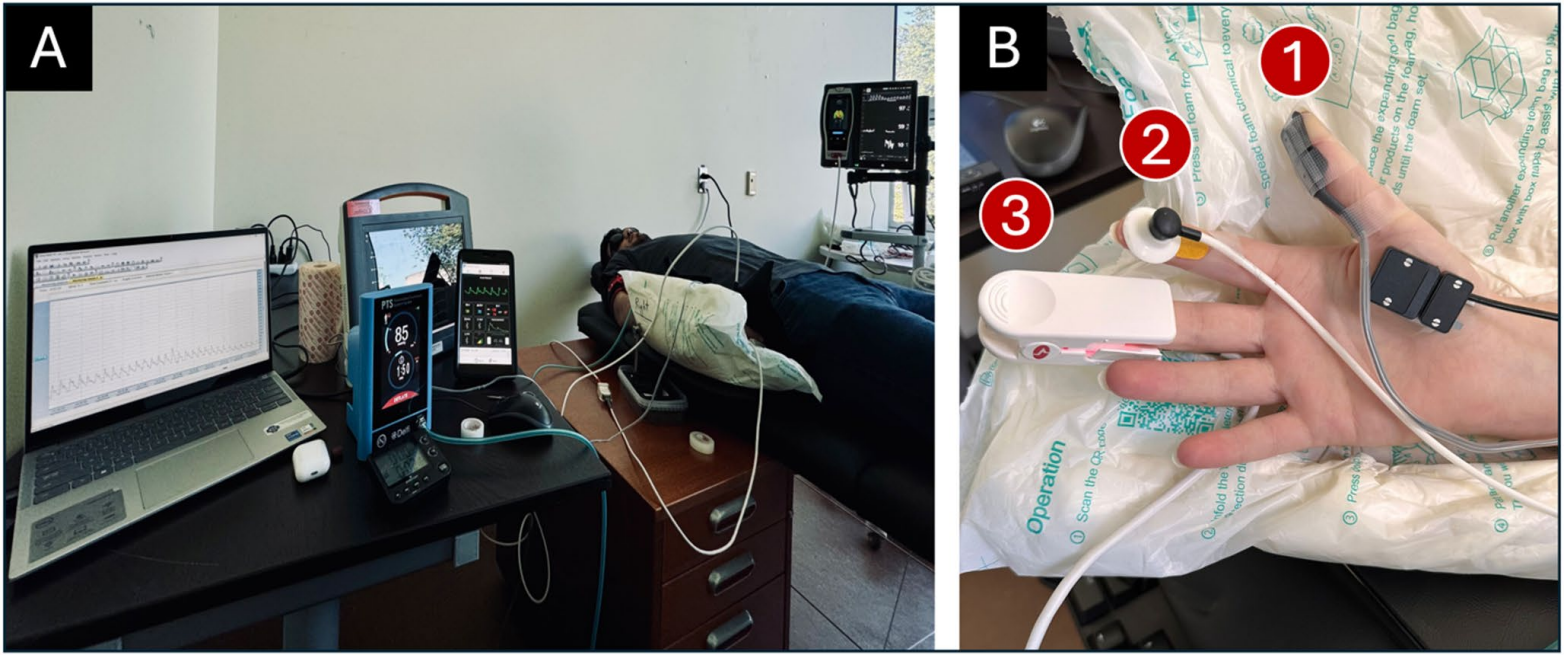
Multisensory configuration for tissue perfusion monitoring. **(A)** Experimental setup during participant monitoring. **(B)** Placement of sensors: the NIRS sensor (**1**) on the thumb, the LDF sensor (**2**) on the index finger, and the PPG pulse oximeter sensor (**3**) on the middle finger. This arrangement enables simultaneous acquisition of NIRS-derived hemodynamic signals, microvascular blood Flux, arterial oxygen saturation, and perfusion index from anatomically adjacent sites

The complete hardware layout is illustrated in Fig. 1. In this experiment, three fingertips on the right hand were used to monitor three adjacent similar tissues with NIRS, along with LDF and PPG pulse oximeter reference sensors. A high-power LDF monitor and probe (VMS-LDF1-HP; Moor Instruments, Axminster, UK) was affixed to the index fingertip, providing continuous, high-temporal-resolution measurements of Flux at 40 Hz in arbitrary perfusion units (AU). To obtain arterial reference parameters, a finger-clip pulse oximeter sensor (MightySat; Masimo Corporation, Irvine, California, USA) was secured to the middle fingertip, delivering the manufacturer’s proprietary PI at 0.5 Hz. A custom-built, continuous-wave (CW) NIRS sensor in a spatially resolved configuration, developed to monitor free tissue transfer (FTT) surgical flaps [36], was positioned on the thumb fingertip to record tissue oxygenation and hemodynamics at a 64 Hz sampling rate.

### Experimental protocol

All sessions were conducted in the same laboratory room equipped with building temperature control, which consistently maintained the ambient temperature near 22 °C. Each recording session was structured to impose two well-controlled ischemic challenges on the participant’s right upper limb, thereby permitting a detailed characterization of the resulting perfusion dynamics. After instrumentation and initiation of each acquisition system, participants remained supine and rested quietly for several minutes while signal quality was verified; the baseline window was started only once signals from all sensors had stabilized (that is, after the initial post-start drift resolved), rather than after a fixed settling time. The protocol began with a five-minute baseline measurement while participants, already lying on their backs and relaxed, remained in that position throughout. Immediately after baseline, a phase of complete ischemia was induced. A digital pneumatic tourniquet (Delfi PTS, Delfi Medical, Vancouver, Canada) encircling the upper arm was inflated to 200 mmHg and maintained at that pressure for two minutes, thereby fully occluding arterial inflow. Longer supra-systolic occlusions (5 min) are often used when the goal is to maximize and standardize the ischemic stimulus and the reactive hyperemia response [37,38]. However, preset-duration vascular occlusion test (VOT) protocols can exhibit substantial inter-individual variability in deoxygenation rate and end-occlusion oxygenation nadir, which also depend on measurement configuration (e.g., site and probe spacing) [39,40]. Therefore, in the present work, a 200-mmHg occlusion was used as a tolerable, repeatable perfusion-suppression step for comparative validation of perfusion-sensitive indices, and oxygenation nadir/deoxygenation kinetics were not treated as study endpoints [41,42]. Once the cuff was released, a ten-minute period of reperfusion followed. This interval captured the expected reactive hyperemic response and provided a window for quantifying flow-mediated recovery. The second challenge started with partial ischemia. Here, the tourniquet was reinflated to 100 mmHg, a level sufficient to restrict venous return but not to stop arterial inflow, thereby causing venous congestion. This partial occlusion was sustained for three minutes, after which the cuff was deflated for a further ten-minute reperfusion period. The final reperfusion phase allowed us to observe how perfusion parameters returned toward baseline following a less severe but longer blockage.

This sequence, comprising baseline, full occlusion, maximal reperfusion, partial occlusion, and a second low-level reperfusion, established distinct hemodynamic states that enabled a comprehensive assessment of microvascular responsiveness.

### Data processing and analysis

To derive the RTPI, we analyzed the THb signal from the near channel of our miniaturized NIRS sensor (source-detector separation = 10 mm), which was selected to bias sensitivity toward superficial microvascular dynamics relative to more prolonged separations [43]. Because photon transport in tissue is depth-weighted and depends on optical properties and probe geometry rather than on a single geometric path [43,44], depth-sensitivity profiles and sampled volumes are inherently different across NIRS, LDF, and PPG [21]. Therefore, the 10 mm channel reduces (but does not eliminate) sampling-depth disparity with superficial optical perfusion references, and cross-modality agreement is interpreted primarily in terms of concordant time-locked dynamic responses to the cuff perturbations rather than identical sampled vascular compartments.

It should be noted that simply subtracting the biological zero from the standard measured LDF Flux can underestimate net perfusion and requires a correction model to be valid [45]; therefore, the biological zero was not subtracted in the present analysis.

From the THb waveform, we extracted three physiologically meaningful features, each chosen to emphasize a distinct physiological aspect of microvascular perfusion:

1. **Pulse-amplitude ratio (PAR)**.

**Fig. 2:**
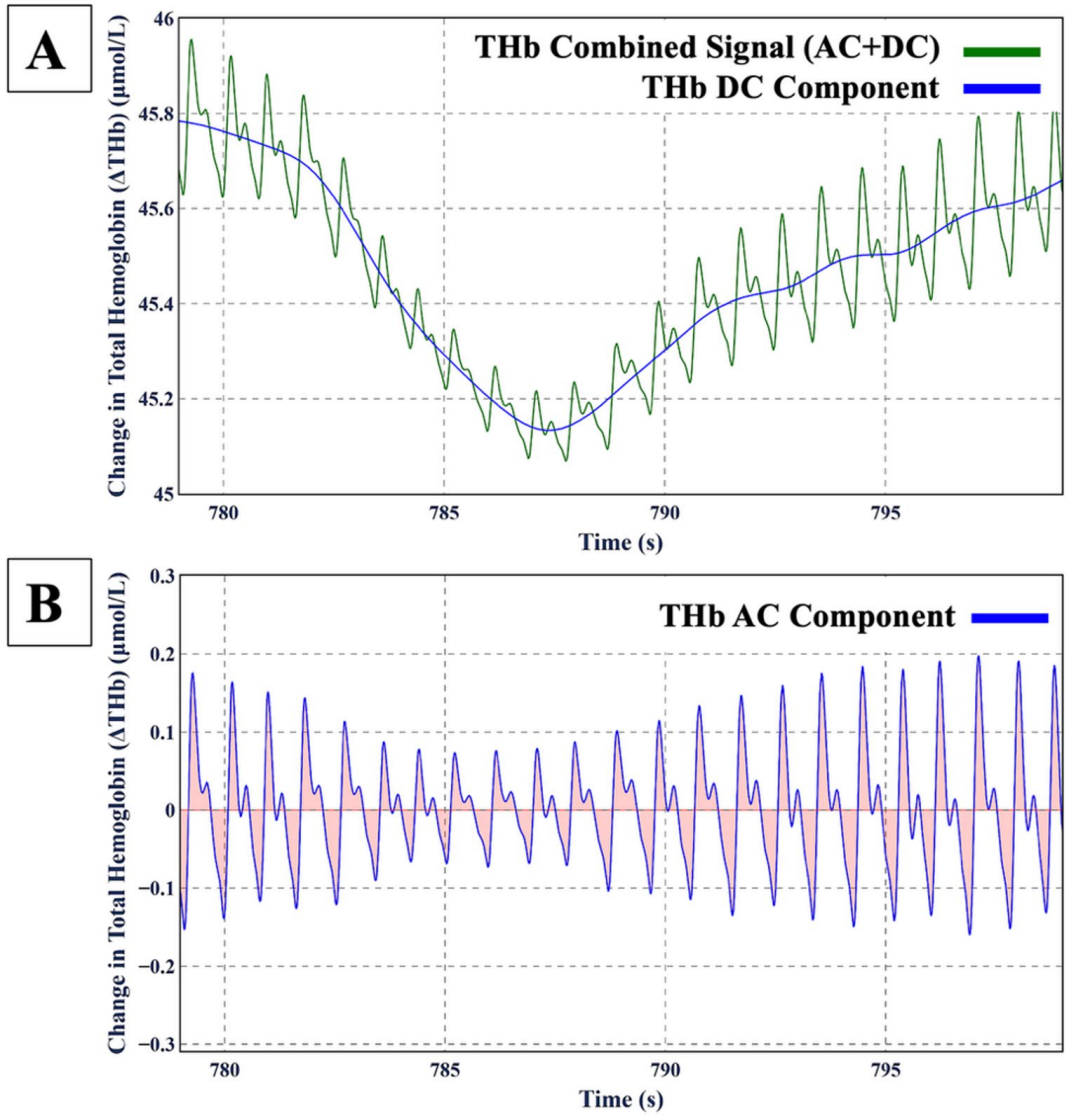
Decomposition of total hemoglobin (THb) signal into AC and DC components. **(A)** Time-series plot showing the raw THb signal (green line) containing both AC and DC components, and the extracted DC component (blue line) obtained using a second-order Butterworth low-pass filter with a cutoff frequency of 0.5 Hz. The DC component represents the signal’s slowly varying baseline. **(B)** The isolated AC component (blue line) was extracted using a second-order Butterworth bandpass filter (0.5–5 Hz), with the red shaded area representing the area under the AC curve. Data shown for a 20-second window from a representative measurement

PAR quantifies the relationship between the pulsatile component of the hemodynamic signal and its slower baseline drift, providing a sensitive measure of microvascular perfusion dynamics. The THb signal was decomposed into alternating current (AC) and direct current (DC) components using frequency-based digital filtering: the pulsatile AC component (cardiac-cycle oscillations) was isolated using a second-order Butterworth band-pass filter (0.5–5 Hz), while the slowly-varying DC baseline was extracted using a second-order Butterworth low-pass filter (0.5 Hz cutoff). The Butterworth filter, which is the default in many recent NIRS pipelines, offers a maximally ripple-free response pass-band, thereby preserving both the low-frequency baseline and the high-frequency cardiac morphology of the NIRS waveform while minimizing phase and amplitude distortion when implemented in a zero-phase configuration [46]. Figure 2A illustrates the decomposition of the THb signal into its constituent components, displaying both the combined signal and the extracted DC component, while Fig. 2B presents the isolated AC component.

Peaks and valleys in the AC component were identified using physiologically constrained detection parameters, including a minimum interpeak distance of 0.25 s and a maximum distance of 2 s, with height, width, and prominence thresholds empirically optimized for reliable feature detection. For each peak, the vertical distance to its immediately subsequent valley was measured and normalized by the DC value interpolated at the temporal midpoint between the peak and valley, yielding an AC/DC (pulse-amplitude) ratio. In PPG, an optical technique that shares fundamental principles with NIRS, the same AC/DC metric (PI) has been validated as a bedside marker of peripheral blood flow and vasomotor tone [47].

PAR was calculated within non-overlapping 2-second windows to ensure sufficient cardiac cycles for reliable estimation while maintaining temporal resolution. Within each window, all normalized peak-to-valley amplitudes were averaged to produce a single PAR value. Windows without detected peaks above the detection threshold were assigned PAR = 0 to indicate the physiologically relevant lower perfusion state of ‘no detectable pulsatility’ and to avoid selection bias that could occur if low-perfusion epochs were excluded from analysis. Because PAR is later used as a time-resolved feature, it is better to maintain continuity during these low-perfusion periods rather than treating them as missing data, which would require interpolation or imputation and might artificially inflate perfusion estimates during occlusion.

2. **Area under the AC curve (AUAC)**.

While PAR quantifies the relative amplitude of cardiac pulsations, AUAC captures the integrated pulsatile signal over a cardiac cycle and can be interpreted as a proxy for beat-to-beat changes in pulsatile blood volume. To ensure physiologically meaningful values and reduce susceptibility to noise and artifacts, AUAC was calculated exclusively for validated cardiac peaks. Within each 2-second analysis window, peaks were identified using the same detection criteria as for PAR. For each confirmed peak, the area under the positive portion of the AC waveform was computed, excluding negative deflections to avoid spurious contributions from signal asymmetry. The trapezoidal rule was applied within the temporal bounds of each peak to yield a robust estimate of cumulative blood volume changes associated with individual cardiac cycles. Area-under-the-curve metrics are physiologically meaningful: in computed tomography (CT) perfusion imaging, cerebral blood volume is derived from the area under a parenchymal contrast-enhancement curve normalized to an arterial curve [48], and the area under the PPG waveform has been reported to relate to stroke volume and may reflect short-term peripheral perfusion changes in healthy volunteers [49].

3. **Derivative (DRV)**.

**Fig. 3:**
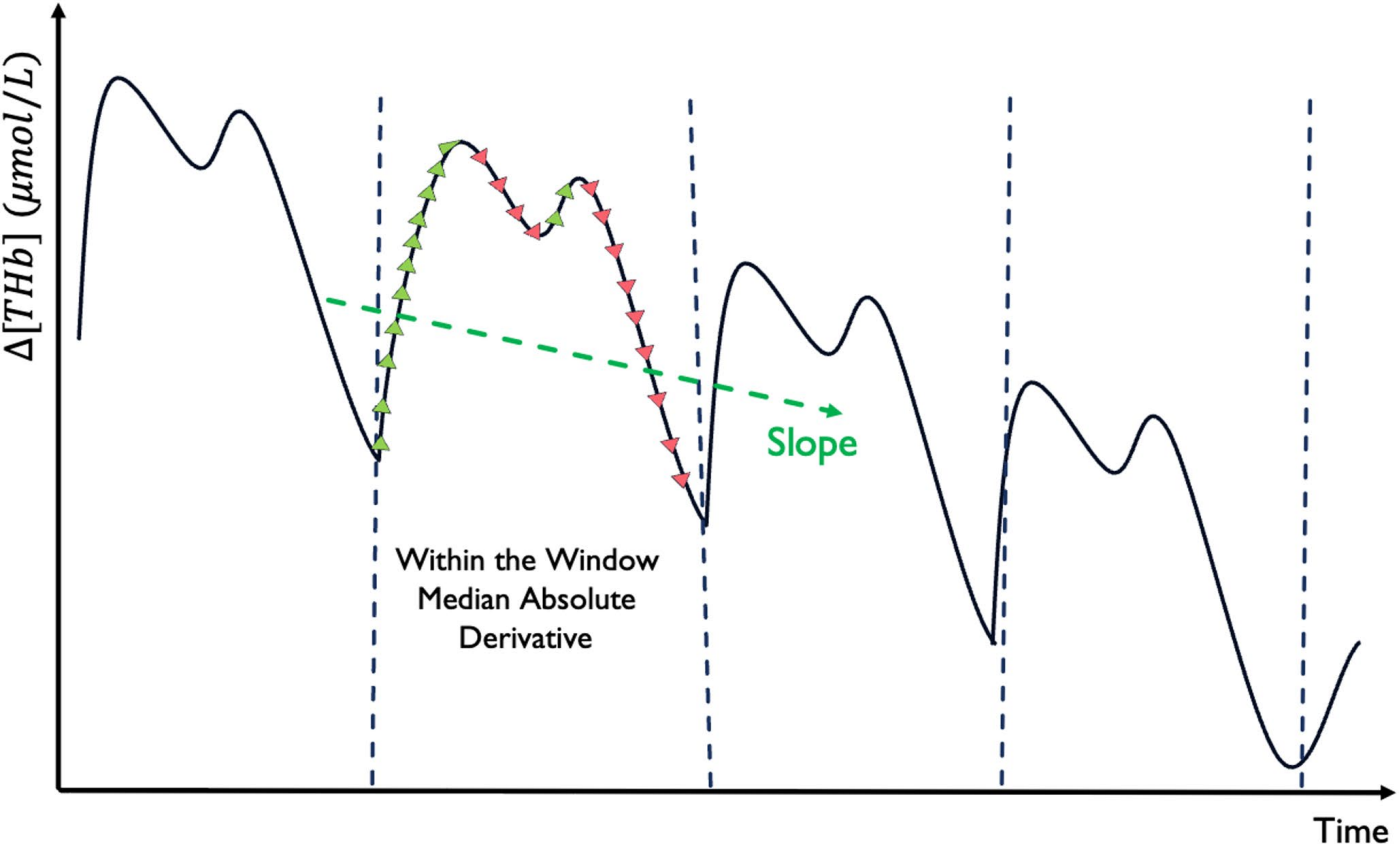
Schematic of DRV calculation within a window and its distinction from a window-level slope. The THb waveform is segmented into non-overlapping windows (vertical dashed lines). Within a window, the first derivative is computed at every sample using finite differences, $\:{d}_{i}=\varDelta\:TH{b}_{i}∕\varDelta\:{t}_{i}$ (colored markers), then converted to the magnitude $\:\left|{d}_{i}\right|$ so that rising and falling portions of the cardiac waveform contribute positively and do not cancel. DRV is obtained by summarizing $\:\left|{d}_{i}\right|$ the within-window signal using a robust statistic (median), yielding a phase-invariant measure of within-window pulsatile activity that does not require cardiac-cycle alignment. The green line illustrates the window-level linear trend (“slope”), which primarily captures net monotonic drift over the window and is therefore conceptually different from DRV

DRV quantifies within-window hemodynamic activity by measuring the magnitude of rapid THb fluctuations (Fig. 3). The raw THb waveform was segmented into non-overlapping 2‑s windows. Within each window, the discrete first derivative was computed at every sample as $\:{d}_{i}=\varDelta\:{\mathrm{T}\mathrm{H}\mathrm{b}}_{i}/\varDelta\:{t}_{i}$, converted to magnitude $\:\left|{d}_{i}\right|$, and summarized as a single robust value using the median: $\:\mathrm{D}\mathrm{R}\mathrm{V}=\mathrm{m}\mathrm{e}\mathrm{d}\mathrm{i}\mathrm{a}\mathrm{n}\left(\left|{d}_{i}\right|\right)$. This definition prevents cancellation between the upslope and downslope portions of the cardiac waveform (which would occur if a signed derivative were averaged) and does not require aligning cardiac cycles or selecting specific points in the cardiac cycle. Importantly, DRV differs from a window-level regression slope: instantaneous derivatives reflect both slow baseline changes and rapid pulsatile dynamics, whereas a fitted slope primarily captures net monotonic drift because the oscillatory component contributes little when averaged over multiple cardiac cycles. Because DRV uses derivative magnitude and a robust summary (median), it is dominated by pulsatile activity and can decrease during hypoperfusion when pulsations flatten, even if the mean THb level rises slowly due to venous pooling. First-derivative (slope) metrics are perfusion-relevant because the NIRS-derived reperfusion slope correlates strongly with Doppler-ultrasound peak blood flow during post-exercise hyperemia [50], and moving-slope features from the first and second derivatives of the PPG waveform have been used to estimate mean arterial pressure from simultaneously recorded arterial blood pressure signals [51], a commonly monitored bedside surrogate of systemic perfusion pressure.

Together, PAR, AUAC, and DRV create a multidimensional representation that comprehensively characterizes tissue perfusion dynamics.

During post-processing, the THb, PI, and Flux signals were first aligned to their respective software-derived timestamps. To ensure temporal coherence across modalities, all three signals were then cross-aligned using a physiologically distinct landmark, the abrupt onset of reperfusion following complete ischemia. While the temporal offset between Flux and THb was minimal (≤ 0.5 s in most cases), likely reflecting slight discrepancies in manual acquisition timing, the PI signal consistently lagged by at least 16 s prior to cross-alignment. PI delay, previously reported in the literature [52], likely results from the signal enhancement algorithm applied to the PPG waveform. This lag was corrected during cross-alignment. Each windowed feature vector was standardized using a robust scaling approach, which centers the data by subtracting the median and scales it by the interquartile range. Unlike traditional z-score normalization, this method down-weights the influence of outliers, thereby reducing the impact of motion artifacts and transient sensor dropouts. Importantly, it places features on comparable scales, allowing each feature to contribute meaningfully to the composite index without being dominated by differences in magnitude.

The scaled triplet entered a principal-component analysis (PCA). Because PCA creates orthogonal axes that capture descending proportions of the variance in the scaled feature triplet, PC1 represents the single linear combination of PAR, AUAC, and DRV that explains the greatest proportion of total variance. We treat this PC1 score as an unsupervised composite and refer to it as the RTPI derived by PCA throughout. For each visit, we computed two accuracy metrics: the Pearson correlation coefficient (r) and the root-mean-squared error (RMSE). Correlation coefficients were first converted to their Fisher-transformed values (z = arctanh r), and these Fisher z–transformed correlations were used for group-level pooling and paired t-tests. The Fisher transform stabilizes the variance of r and yields an approximately normal sampling distribution, thereby justifying parametric pooling and the construction of 95% confidence intervals [53]. Moreover, segment-wise pooled Pearson correlation coefficients with 95% confidence intervals were computed for three pairings: RTPI vs. PI, PI vs. Flux, and RTPI vs. Flux.

To quantify session-to-session change, we applied a two-tailed paired-sample Student’s t-test comparing the Fisher z values from visit 1 and visit 2 within participants. A Shapiro–Wilk test did not indicate a departure from normality, so no non-parametric substitute was required. Reliability across visits was evaluated using the intraclass correlation coefficient (ICC) model 3,1, a two-way mixed-effects, single-measurement coefficient. This approach is recommended when the same fixed measurement occasions (visit 1 and visit 2) are compared and when the focus is on consistency rather than absolute agreement [54].

Individual Fisher-transformed correlation coefficients (Fisher z values) were combined across sessions (participant × visit) using a weighted Stouffer Z approach [55,56]. Each session’s z-score was weighted by √(*n* − 3), where n is that session’s window count, to reflect the approximate precision of Fisher z. The weighted sum was then divided by the square root of the sum of squared weights to yield a weighted Stouffer Z, which was converted to a two-sided omnibus p-value using a standard normal approximation [57]. This procedure produces a single omnibus p-value that reflects the aggregate evidence for association across the cohort while accounting for differences in window counts across sessions.

**Fig. 4:**
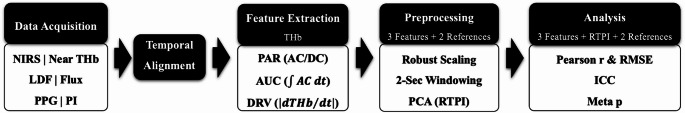
**Pipeline from acquisition to analysis for RTPI and reference comparisons.**

To test whether the PCA-derived index correlates more strongly with each reference signal than any individual feature, we compared pairs of dependent correlations that share a common variable using the Williams test for overlapping correlations [58,59]. We use the term Williams test to follow standard nomenclature and documentation commonly used in practice, for example, the materials that catalogue and implement the Williams and Steiger procedures in the cocor package in R software [60]. For each session (participant × visit), we calculated one-sided p-values to test if the correlation between RTPI (PCA) and the reference signal exceeded the correlation between each feature and the reference signal, after accounting for the intercorrelation between RTPI (PCA) and the features. Because inference was carried out per session, we combined the resulting one-sided p-values across sessions (participant × visit) by first converting them to standard normal deviates and then using a weighted Stouffer Z with weights √(*n* − 3), as above, to obtain a single directional group p-value for each feature-to-reference comparison. Figure 4 summarizes the pipeline from data acquisition to RTPI construction and statistical evaluation.

To evaluate whether the preceding complete occlusion introduced residual effects that could bias subsequent partial-ischemia analyses, baseline comparability immediately before the 100-mmHg ischemia phase was assessed using protocol-defined reference windows rather than reference-modality features. Using within-period baselines to diagnose and mitigate carryover is a standard strategy in repeated-measures and crossover designs [61]. For each visit, two end-of-segment windows were extracted: (i) baseline-end, defined as the last 120 s (2 min) of the baseline segment, and (ii) pre-partial, defined as the last 120 s (2 min) of Recovery 1 before cuff inflation to 100 mmHg. Because multiple visits per participant are not statistically independent, visit-level values were summarized at the participant level before inference to avoid pseudo-replication [62].

For each metric (RTPI, Flux, and PI), a carryover difference was computed per visit as Δcarryover = mean (pre-partial) – mean (baseline-end). Baseline-end and pre-partial values were evaluated using equivalence testing rather than relying on a non-significant difference test, since a non-significant result does not establish equivalence [63]. Equivalence was tested using the two one-sided tests (TOST) procedure [64]. A smallest-effect-size-of-interest (SESOI) equivalence margin (±δ) was prespecified on each metric’s native scale as δ = 0.2 × SD_0_, where SD_0_ was the median within-window baseline-end standard deviation across visits, linking the decision threshold to typical measurement variability [65,66]. The 0.2 multiplier corresponds to a conservative “small” standardized difference threshold [67]. In parallel, to check whether Recovery 1 had reached a quasi-steady state immediately before the partial-ischemia phase, the linear trend (slope) over the pre-partial window was computed per visit and summarized at the participant level; recovery kinetics and reoxygenation slopes are commonly used summaries of occlusion-test responses in vascular function assessments [68,69].

To evaluate RTPI performance independently of reference modalities, we quantified its ability to discriminate between the controlled physiological states induced by the ischemia-reperfusion protocol, consistent with established responsiveness and known-state validation approaches for evaluative measures [70,71]. For each visit, the RTPI time series was segmented into the five protocol phases (baseline, complete ischemia at 200 mmHg, recovery 1, partial ischemia at 100 mmHg, and recovery 2). Within each phase, segment-level means were computed. Because each participant completed two visits, segment means were computed within each visit and then averaged across the two visits to produce a single participant-level estimate, ensuring that inference was performed on independent participants rather than on repeated sessions [72].

Three within-participant contrasts were pre-specified to probe responsiveness to the protocol perturbations: complete ischemia versus baseline (200 mmHg occlusion minus baseline), partial ischemia versus baseline (100 mmHg occlusion minus baseline), and partial ischemia versus post-complete-occlusion recovery (100 mmHg occlusion minus recovery 1). Paired-sample inference was performed on participant-wise difference scores for each contrast [73]. Contrast magnitude was quantified using Cohen’s d_z_, defined for paired designs as the mean of the within-participant differences divided by the standard deviation, consistent with standardized change metrics used in responsiveness analyses [70,71,73]. Family-wise error across planned contrasts was controlled using Holm’s sequentially rejective multiple-testing procedure [74], and uncertainty in standardized effects was estimated using nonparametric bootstrap resampling of the paired differences (2,000 resamples) [75]. The same contrasts were computed for the reference signals to facilitate contextual comparison, with protocol phase labels defined exclusively by the cuff inflation schedule.

To assess test-retest reliability of RTPI independently of reference modalities, contrast-based reliability was evaluated using ICC (3, 1) calculated from participant-level contrast values across visits. For all participants, each contrast value (e.g., 200 mmHg minus baseline) was computed separately within each visit from segment means and then used as the repeated measure for ICC estimation. This approach evaluates the cross-visit consistency of occlusion response magnitudes while avoiding reliance on absolute signal levels or treating time points or segments as independent observations.

## Results

**Fig. 5:**
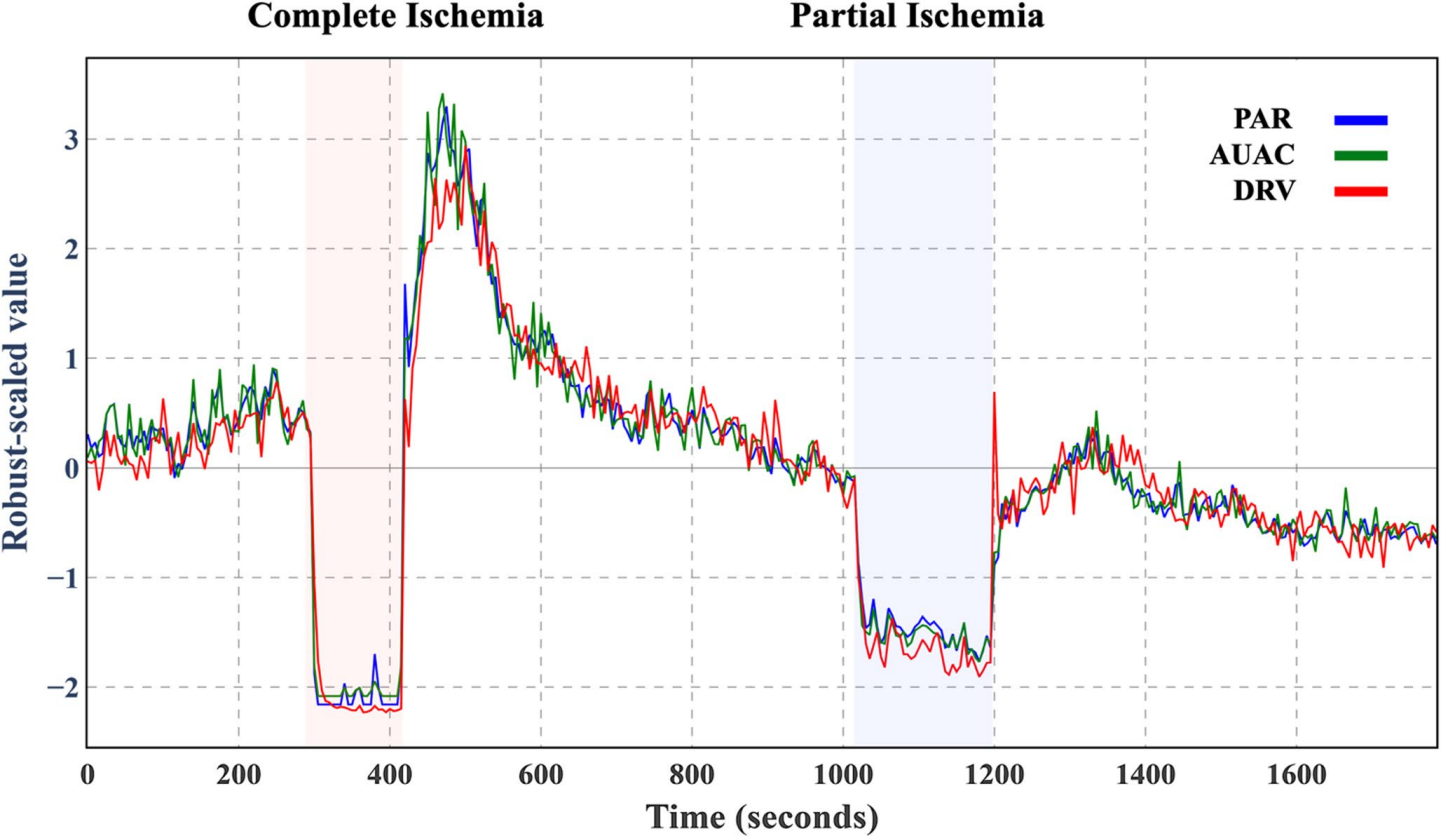
Temporal dynamics of all three features during the entire protocol. Time-series plot showing robust-scaled values of NIRS-derived features: pulse amplitude ratio (PAR), area under the AC curve (AUAC), and first derivative (DRV) over the full 30-minute experimental protocol

All 20 participants completed the ischemia–reperfusion protocol without adverse events. As anticipated, NIRS-derived perfusion features responded predictably: both the 200-mmHg complete occlusion and the 100-mmHg partial occlusion caused immediate, measurable reductions across all features. Subsequent cuff release induced a transient reactive hyperemic response during the recovery phase (Fig. 5).

Across the planned comparisons against Flux and PI, the composite RTPI, which integrates all three hemodynamic features, generally demonstrates a stronger correspondence than any individual feature metric in most comparisons. The session-wise PC1 loadings had mean ± SD values of 0.596 ± 0.028 for PAR, 0.561 ± 0.024 for AUAC, and 0.572 ± 0.032 for DRV. PAR’s slightly larger loading is consistent with PC1 being more aligned with pulsatile, flow-sensitive information, given that PAR is defined as the ratio of pulsatile (AC) to non-pulsatile (DC) components. The near-equal magnitude of the loadings (approximately 0.56 to 0.60) indicates that PC1 is not driven by a single feature but instead reflects a shared latent hemodynamic trajectory integrating pulsatile amplitude (PAR), pulsatile waveform area/energy (AUAC), and derivative-based THb dynamics (DRV). Table 1 presents a statistical validation of NIRS-derived perfusion parameters against PI and Flux. Although the features (PAR, AUAC, and DRV) are derived using distinct mathematical approaches, they exhibited broadly similar overall trends. However, closer examination revealed distinct temporal dynamics for each feature (Fig. 5). Specifically, during recovery from partial ischemia, DRV demonstrated the most pronounced overshoot, whereas after complete ischemia, PAR and AUAC reached higher peaks during reperfusion, while DRV increased more gradually and to a lower maximum.

**Table 1:** Group-level correlation coefficients, intraclass-correlation coefficients, and error metrics for all NIRS-derived parameters versus the two reference modalities

Parameter	Reference	Test-Retest *p*	ICC (95% CI)	Pooled *r* (95% CI)	Meta *p*	RMSE
**PAR**	PI	0.73	0.04 (-0.43, 0.50)	0.85 (0.84, 0.85)	1.17 × 10⁻¹³	0.60
**PAR**	Flux	0.55	0.66 (0.28, 0.86)	0.73 (0.73, 0.74)	2.16 × 10⁻⁸	0.83
**AUAC**	PI	0.66	0.29 (-0.21, 0.66)	0.83 (0.82, 0.83)	6.52 × 10⁻¹²	0.63
**AUAC**	Flux	0.85	0.76 (0.45, 0.91)	0.71 (0.71, 0.72)	2.88 × 10⁻⁸	0.87
**DRV**	PI	0.56	0.06 (-0.42, 0.51)	0.80 (0.80, 0.81)	1.13 × 10⁻¹⁰	0.68
**DRV**	Flux	0.81	0.54 (0.10, 0.80)	0.70 (0.69, 0.70)	3.22 × 10⁻⁷	0.88
**PCA**	PI	0.62	0.10 (-0.39, 0.54)	0.85 (0.84, 0.85)	3.72 × 10⁻¹³	0.60
**PCA**	Flux	0.94	0.70 (0.34, 0.88)	0.75 (0.75, 0.76)	1.39 × 10⁻⁸	0.79
**PI**	Flux	0.63	0.51 (0.06, 0.79)	0.68 (0.68, 0.69)	1.20 × 10⁻⁶	0.93

**Fig. 6:**
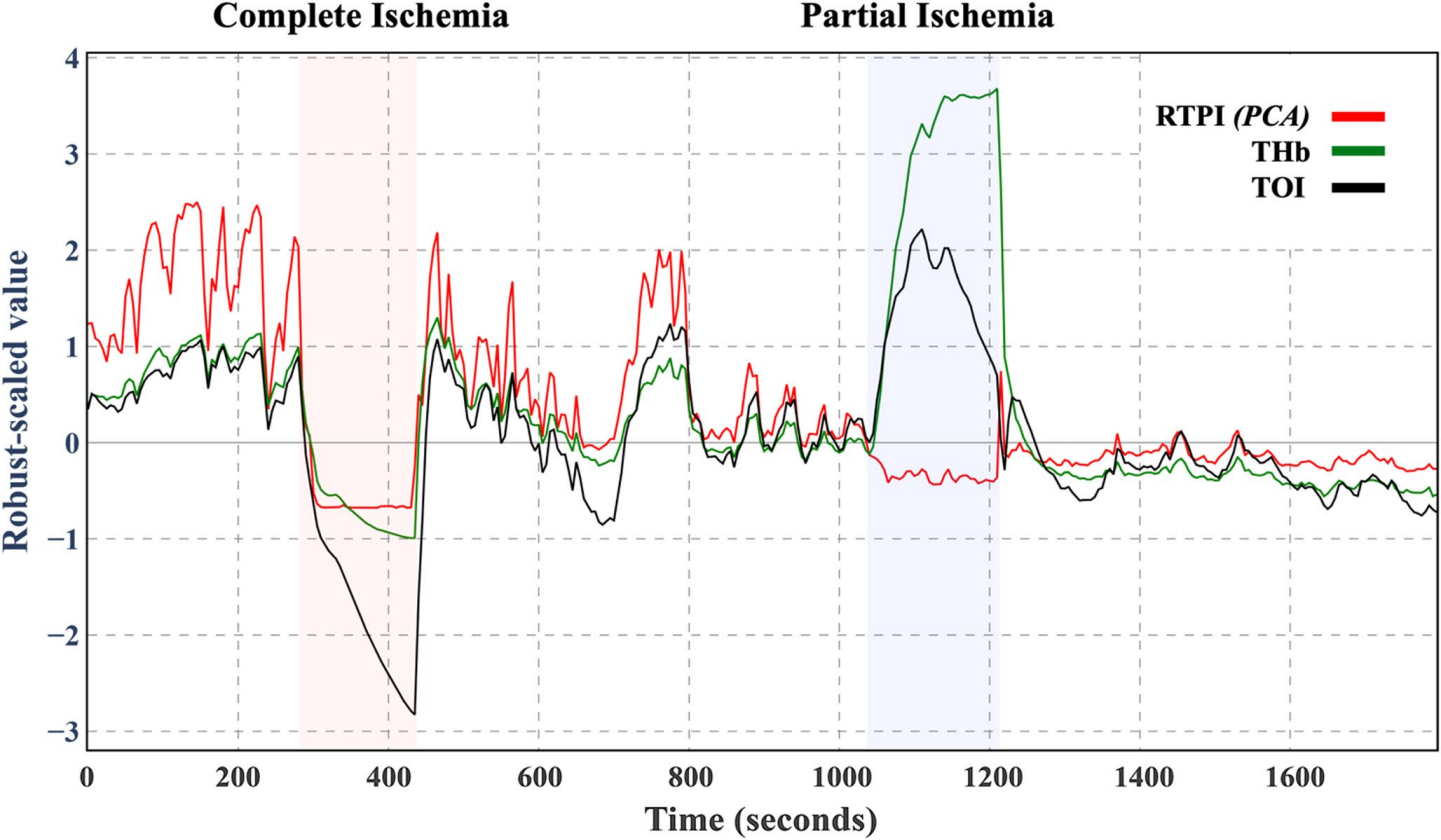
Comparative hemodynamic responses of NIRS tissue perfusion index (RTPI-PCA), THb, and TOI during complete and partial ischemia challenges. Time-series plot showing robust-scaled values during a complete experimental protocol. The red-shaded region indicates complete ischemia induced by inflation of a 200-mmHg cuff, producing immediate and synchronized decreases in all three parameters. The blue-shaded area represents partial ischemia at a 100-mmHg cuff pressure, revealing divergent responses: RTPI (red) demonstrates immediate perfusion compromise, whereas TOI (black) initially increases due to venous congestion before ultimately declining. THb (green) shows pronounced accumulation during partial ischemia, reflecting blood pooling from obstructed venous return

As expected for an index constructed from perfusion-sensitive pulsatile and kinetic features, RTPI responds rapidly to occlusion and cuff release and, in our protocol, detects transition onsets earlier than TOI or THb (Fig. 6), consistent with physiological validation of its perfusion sensitivity.

**Fig. 7:**
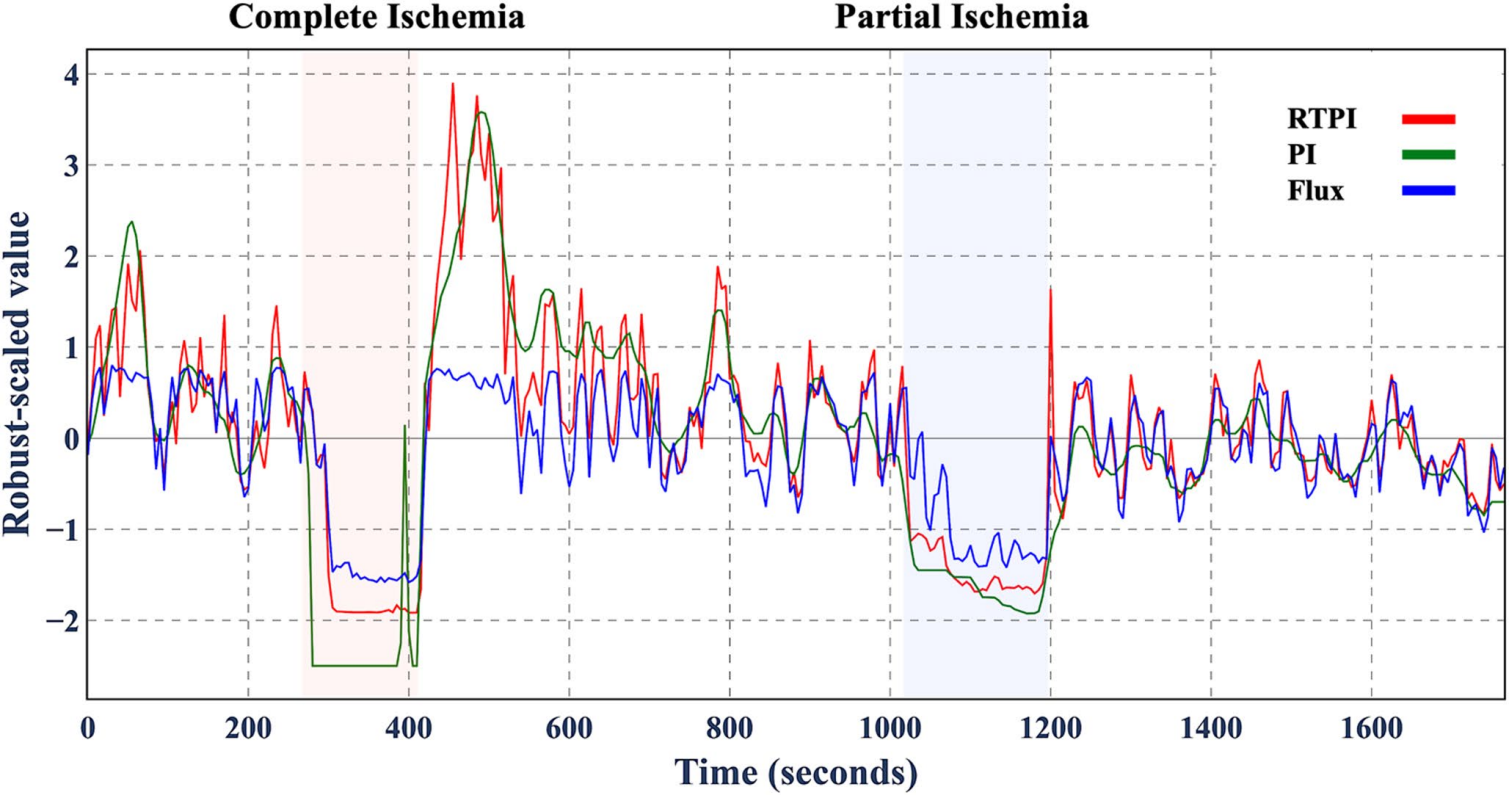
Temporal dynamics of NIRS regional tissue perfusion index (RTPI) compared with reference perfusion measurements throughout the ischemia-reperfusion protocol. Time-series plot showing robust-scaled values of RTPI derived from principal component analysis (red), photoplethysmography perfusion index (PI, green), and laser Doppler Flux (blue) over the complete 30-minute experimental protocol. All three measures demonstrate roughly synchronized responses during complete ischemia and partial ischemia periods, with concordant perfusion cessation and reactive hyperemia upon reperfusion

**Fig. 8:**
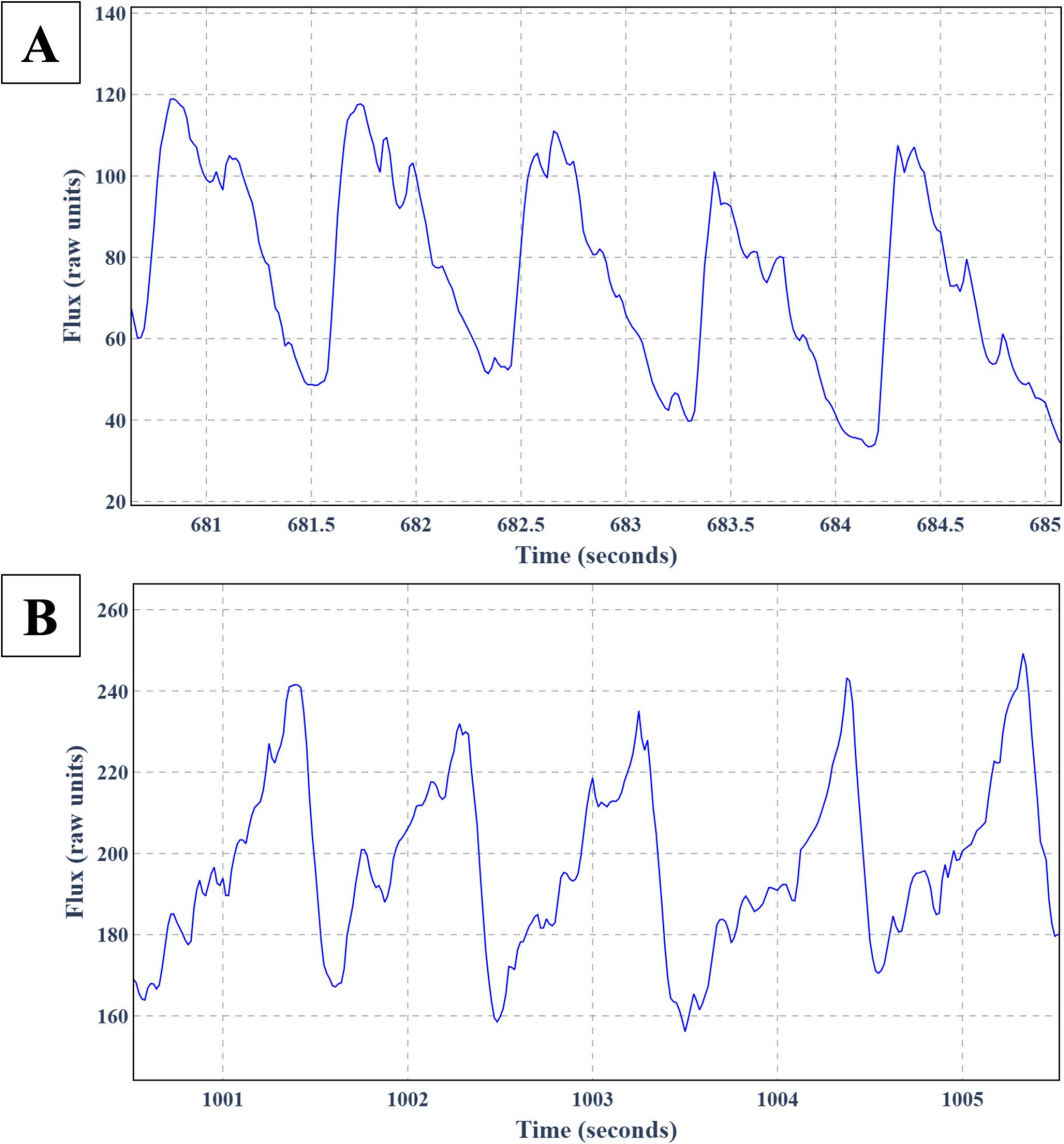
Examples of LDF pulse waveform morphology observed in the dataset. **(A)** typical pulse contour with the systolic peak preceding the dicrotic notch, **(B)** atypical morphology in which a notch-like feature precedes the systolic peak

**Fig. 9:**
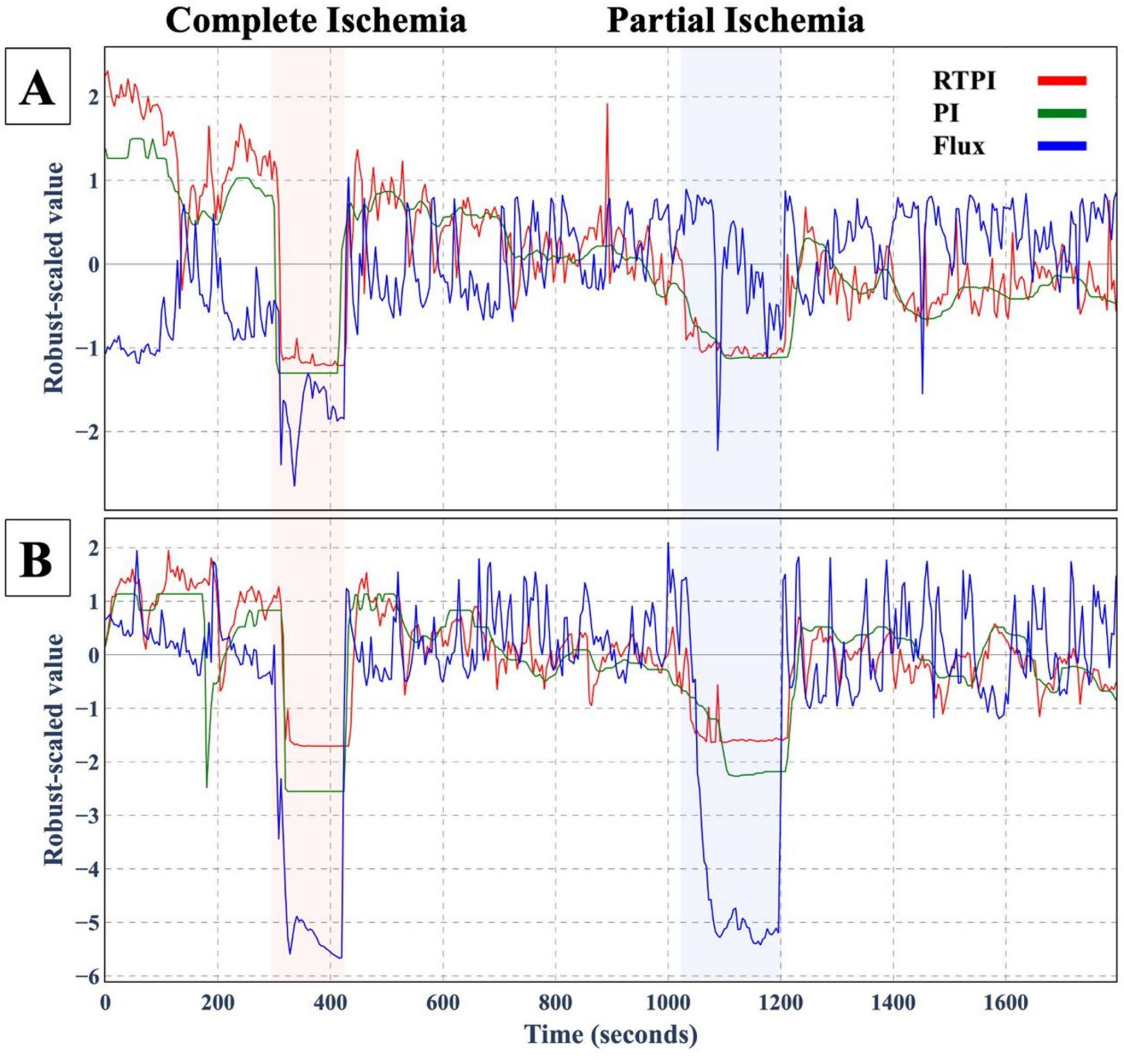
Representative robust-scaled time-series from two sessions of a single participant under the same protocol, showing RTPI (red), PPG PI (green), and LDF Flux (blue) traces. Shaded intervals indicate cuff-inflation phases. **(A)** Session 1 shows minimal net change in LDF Flux during the 100-mmHg occlusion phase, while RTPI and PI show a consistent decrease. **(B)** Session 2 shows a pronounced decrease in LDF Flux during the 100-mmHg occlusion phase, while RTPI and PI again decrease in the same direction, illustrating greater cross-session consistency for RTPI and PI than for LDF in this participant

Although most datasets demonstrated physiologically consistent responses in both RTPI and the reference measurements (Fig. 7), datasets from three participants (across both visits) were excluded from all analyses, and all reported results are based on 17 participants. Two male participants were excluded due to atypical inverted laser Doppler Flux responses, with heartbeat waveforms reversed in polarity (Fig. 8) and a trend in which Flux increased when both PI and RTPI decreased, resulting in very low or negative correlations. These two participants also had the highest body mass indices in the cohort (29 and 31 kg/m²), raising the possibility that greater soft-tissue thickness reduced the effective sampling depth of the fingertip Doppler probe and/or attenuated cuff pressure transmission on the upper arm, thereby altering the achieved degree of vascular occlusion; however, this remains speculative. One female participant’s data were excluded due to severe motion artifacts from frequent hand movement, which affected all recorded channels. Among the retained recordings, several sessions showed limitations in one or both reference modalities. In two sessions (from different participants), the PI signal decreased only after a marked delay following cuff inflation to 100-mmHg. Moreover, in one participant measured across two separate sessions, the LDF response during the 100-mmHg partial occlusion ranged from minimal change to a pronounced decrease, whereas RTPI and PI decreased consistently (Fig. 9).

Paired t-tests on Fisher-transformed correlations showed no significant differences between visits for any parameter-reference combination (all *p* > 0.55), indicating stable performance across repeated measurements. Moreover, Intraclass correlation coefficients (ICC 3,1) revealed varying degrees of reliability across parameters. When compared against Flux, all measures demonstrated good test-retest reliability. However, when validated against PI, most parameters showed poor to fair reliability, with ICC values ranging from 0.04 to 0.29, indicating lower between-visit consistency of the correlation-based association when PI is used as the reference.

A direct comparison between the two reference standards (PI vs. Flux) yielded a lower correlation (*r* = 0.68, 95% CI [0.68, 0.69]) and relatively high error (RMSE = 0.93) compared with NIRS-derived parameters. Moreover, Stouffer’s Z meta-analysis confirmed highly significant correlations for all parameter-reference pairs (all *p* < 0.05). The strongest meta-analytic evidence was observed for the combination of RTPI and PI (*p* = 3.72 × 10^–13^). Additionally, RMSEs were consistently lower when parameters were validated against PI (range: 0.60–0.68) compared to Flux (range: 0.79–0.93). Although the raw correlations suggest that PCA generally aligns most strongly with the references, group-level results from the Williams test for dependent correlations reveal a more nuanced pattern of advantages and exceptions, as summarized in Table 2.

**Table 2:** Group-level comparisons of PCA (RTPI) versus individual features using dependent-correlation tests

Feature	Reference	$\:\varDelta\:\boldsymbol{r}$(PCA minus feature)	Group *p*-value (one-sided)
**PAR**	PI	-0.02	≈ 1.00 *
**AUAC**	PI	0.07	1.29 × 10⁻^262^
**DRV**	PI	0.14	≈ 0 **
**PAR**	Flux	0.03	1.57 × 10⁻^15^
**AUAC**	Flux	0.05	8.32 × 10⁻^35^
**DRV**	Flux	0.10	3.55 × 10⁻^294^

* *p* = 1–1.46 × 10⁻^30^; rounded to 1.0 in double precision

** *p* ≈ 2.00 × 10⁻^524^; underflows to 0.0 in double precision

When PI was used as the reference, PCA outperformed AUAC and DRV (Δr = 0.07 and 0.14, respectively; *p* < 0.05), whereas no gain was observed relative to PAR (Δr = − 0.02, *p* = 1). In contrast, when Flux served as the reference, PCA consistently outperformed all individual features, with gains ranging from Δr = 0.03 to 0.10 and minimal group p values. These findings indicate that the composite PCA-based index provides an advantage over single features in capturing reference-related variance, except when PI is compared with PAR.

Table 3 summarizes pooled Pearson correlations between RTPI, PI, and Flux across protocol segments. Exclusion rows report r recomputed on pooled data after excluding the specified segments (not an average of segment-specific r values). The baseline showed the weakest PI-Flux association (*r* = 0.21) and a modest RTPI-Flux association (*r* = 0.43). Full ischemia exhibited low pooled correlations across pairings (RTPI vs. PI: *r* = 0.31; PI vs. Flux: *r* = 0.32; RTPI vs. Flux: *r* = 0.32). Recovery 1 showed a strong RTPI-PI correlation (*r* = 0.83) and a high RTPI-Flux correlation (*r* = 0.66). When baseline was excluded, pooled correlations increased across all pairings (RTPI vs. PI: *r* = 0.86; PI vs. Flux: *r* = 0.71; RTPI vs. Flux: *r* = 0.76). Across segments, RTPI vs. Flux generally exceeded PI vs. Flux, except during full ischemia (equal) and partial ischemia (nearly identical).

**Table 3:** Pooled Pearson correlation coefficients (r) with 95% confidence intervals (CI) between RTPI and PI, PI and Flux, and RTPI and Flux across experimental segments

Segment	RTPI vs. PI Pooled *r* (95% CI)	PI vs. Flux Pooled *r* (95% CI)	RTPI vs. Flux Pooled *r* (95% CI)
**Baseline**	0.60 (0.58, 0.62)	0.21 (0.18, 0.24)	0.43 (0.40, 0.45)
**Full Ischemia**	0.31 (0.24, 0.38)	0.32 (0.26, 0.35)	0.32 (0.27, 0.36)
**Recovery 1**	0.83 (0.82, 0.83)	0.55 (0.54, 0.57)	0.66 (0.65, 0.67)
**Partial Ischemia**	0.62 (0.59, 0.64)	0.57 (0.55, 0.60)	0.56 (0.54, 0.59)
**Recovery 2**	0.65 (0.63, 0.66)	0.55 (0.53, 0.56)	0.67 (0.66, 0.69)
**Baseline excluded**	0.86 (0.86, 0.87)	0.71 (0.71, 0.72)	0.76 (0.76, 0.77)
**Baseline and Full Ischemia Excluded**	0.84 (0.84, 0.84)	0.64 (0.63, 0.65)	0.72 (0.71, 0.72)

Carryover between the complete-occlusion and partial-occlusion provocations was assessed by comparing the baseline-end window with the pre-partial window and by evaluating end-of-Recovery 1 stability. These analyses indicated that baseline-end and pre-partial values were not equivalent for Flux, PI, or RTPI, although end-of-Recovery 1 trends (slopes) were near zero on average. Accordingly, partial-ischemia discrimination was evaluated using both baseline and Recovery 1 as reference states. Detailed carryover and stability results are provided in Online Resource 1 (Tables S1–S2).

Protocol-state discrimination analysis using protocol-defined phase labels (rather than reference-modality correlations) showed that RTPI decreased significantly during both occlusion phases relative to baseline and recovery. During partial ischemia (100 mmHg), RTPI exhibited the largest standardized change (Cohen’s d_z_ = − 5.02), whereas during complete ischemia (200 mmHg), Flux showed a slightly larger standardized change (d_z_ = − 4.18) than RTPI (d_z_ = − 3.59). All planned contrasts remained significant after Holm correction (all p_Holm_ ≤ 3.32 × 10⁻⁶). Full contrast statistics are provided in Online Resource 1 (Tables S3–S5).

Test–retest reliability of contrast responses across visits was highest for RTPI, with moderate ICCs for complete ischemia minus baseline (ICC = 0.467) and partial ischemia minus baseline (ICC = 0.390), and lower reliability for partial ischemia minus recovery 1 (ICC = 0.235). In contrast, PI showed low ICCs across contrasts (ICC = 0.073 to 0.136), while Flux exhibited poor reliability, including negative ICC estimates, indicating substantial within-participant variability across sessions relative to between-participant differences (Table 4). Confidence intervals were wide for all measures, consistent with limited precision in reliability estimation under the available sample size and visit structure. These results suggest RTPI offers greater cross-session consistency than the reference modalities for protocol-defined occlusion contrasts, although reliability still varies by contrast, and no metrics achieved high reliability.

Together, these analyses evaluated (i) responsiveness to known perfusion states and (ii) cross-visit repeatability of occlusion responses, independent of any reference modality.

**Table 4:** Test–retest reliability of occlusion responses

Metric	Test–retest ICC of mean contrast: Full Ischemia − Baseline (95% CI)	Test–retest ICC of mean contrast: Partial Ischemia − Baseline (95% CI)	Test–retest ICC of mean contrast: Partial Ischemia – First Recovery (95% CI)
**PI**	0.136 (-0.543, 0.707)	0.132 (-0.546, 0.705)	0.073 (-0.586, 0.674)
**RTPI**	0.467 (-0.234, 0.849)	0.390 (-0.321, 0.820)	0.235 (-0.467, 0.755)
**Flux**	-0.525 (-0.869, 0.160)	-0.048 (-0.660, 0.602)	-0.003 (-0.633, 0.630)

## Discussion

This study introduces a novel, non-invasive near-infrared spectroscopy framework for quantifying and continuously monitoring regional tissue perfusion. By extracting three physiologically meaningful features, PAR, AUAC, and DRV, from the NIRS-derived THb signal and combining them using principal component analysis, we derived a composite Regional Tissue Perfusion Index (RTPI).

The RTPI demonstrated acceptable concurrent validity with established reference standards, yielding a correlation of *r* = 0.85 with the photoplethysmography-based perfusion index and *r* = 0.75 with laser Doppler Flux. These findings support the feasibility of NIRS-based perfusion tracking with clinically relevant fidelity. Furthermore, each individual feature showed statistically significant correlations with both references, reinforcing their standalone value as interpretable perfusion surrogates. Notably, the strong association between PAR and PI is consistent with their mutual reliance on pulsatile-to-baseline amplitude ratios (AC/DC), a well-characterized parameter in optical hemodynamic monitoring [25,76].

Although the three NIRS-derived features exhibited concordant trends during hemodynamic transitions, subtle differences during ischemia and reperfusion phases highlighted their physiological complementarity. PAR and AUAC primarily reflect the reappearance of pulsatile flow, whereas DRV quantifies the instantaneous rate of change in hemoglobin concentration, providing temporal resolution independent of cardiac cycles. By integrating these features via PCA, we derived the composite RTPI, which slightly improved correspondence with both PI and laser Doppler Flux relative to individual features (except PAR when PI was the reference), while maintaining a fully reference-free architecture.

Beyond incremental performance improvements, PCA confers several methodological advantages. First, it addresses a persistent challenge in pulse peak detection. Situations such as motion artifacts, low-amplitude waveforms during hypoperfusion [77], clipped peaks from sensor saturation [78], or waveform variability due to arrhythmia or hemodynamic instability can result in zero or missing peak-based features [79]. PCA mitigates this issue by redistributing signal variance across components, preserving signal continuity even when individual feature channels degrade. In our analysis, substantial inter-participant differences in pulse amplitude required optimization of the findpeaks parameters to reliably detect true peaks while rejecting noise. This variability could limit the extent to which a single fixed parameter set generalizes to prospective studies or independent cohorts. This challenge may have motivated the development of advanced solutions in earlier works (e.g., using an ensemble of algorithms [80] or Masimo SET [81]) and underscores that, while peak-based features (e.g., PAR) can achieve higher accuracy, their reliable extraction often requires sophisticated algorithms and intensive processing. Second, PCA has a well-established track record in enhancing NIRS signal fidelity. By emphasizing high-variance components, PCA can reduce noise and certain physiological confounders, with prior work reporting performance comparable to Kalman and wavelet-based approaches in specific applications [82,83]. Third, PCA’s efficiency in computation, due to its minimal processing demands, and its unsupervised approach make it particularly suitable for real-time use in clinical environments, where signal characteristics can vary unpredictably and reference modalities are often absent. Finally, the unsupervised nature of PCA also allows it to extract orthogonal components purely from the covariance structure of the NIRS features, eliminating reliance on external reference signals and thus avoiding the propagation of motion artifacts and site-specific variability that frequently degrade laser-Doppler Flux measurements [84,85,86]. These characteristics make PCA particularly well-suited for real-time clinical applications, where signal properties may vary unpredictably across individuals or conditions.

The stronger correlation observed between NIRS-derived parameters and PI, relative to laser Doppler Flux, likely reflects fundamental differences in measurement principles. NIRS and PPG are absorption-based optical methods sensitive to pulsatile hemodynamics; NIRS estimates changes in hemoglobin concentration, whereas PPG primarily reflects pulsatile blood volume variations. In contrast, laser Doppler flowmetry derives an index of microvascular perfusion from Doppler frequency shifts induced by moving red blood cells, with the output proportional to both red blood cell velocity and concentration. Because NIRS and PPG lack an explicit velocity dimension, they may not capture certain dynamic aspects of flow that are accessible to LDF-based measurements.

The correlation between the two reference standards themselves (*r* = 0.68) provides crucial context for interpreting our validation results. This inter-reference disagreement inherently limits the achievable correlation for any derived parameter, because no measurement can simultaneously achieve perfect agreement with two references that disagree. This mathematical constraint establishes both a performance ceiling and realistic expectations for NIRS-derived parameters. Remarkably, the NIRS-derived RTPI achieved correlations with individual references that exceeded the correlation between the references themselves. This finding not only validates our approach but also suggests that RTPI captures distinct perfusion information that aligns more closely with each reference modality than the references align with one another.

The low correlation observed between the metrics under baseline conditions and under full ischemia is physiologically plausible. Before cuff inflation, there is no shared perturbation acting on all modalities, so RTPI, PI, and Flux are not forced to change together; each is dominated by local microvascular variability. Baseline skin blood flow is known to be spatially heterogeneous and to show spatiotemporal variability even at rest [87]. In addition, spontaneous microvascular oscillations can be relatively stochastic with limited spatial coherence compared with imposed physiological challenges [88]. When baseline variability is small, Pearson correlation is also expected to be attenuated by restricted range, especially in the presence of measurement noise; small “wiggles” make unshared local fluctuations and measurement error a larger fraction of the observed signal, reducing shared covariance and therefore reducing r [88]. Furthermore, during complete ischemia, LDF approaches the biological zero condition, where a substantial component of the signal reflects Brownian-motion-related contributions from the interstitial compartment rather than true perfusion [89]. Under these near-zero-flow conditions, residual LDF fluctuations can become a larger fraction of the total output, further reducing apparent coupling with perfusion-linked indices such as RTPI and PI.

The signal irregularities observed in a subset of sessions underscore known limitations of the two clinical reference standards. Specifically, during the 100-mmHg partial (venous) occlusion, Flux exhibited attenuated and between-session inconsistent changes in one participant in this dataset, whereas RTPI and PPG-derived PI decreased consistently. This is not interpreted as a universally “incorrect” physiological response, because LDF quantifies microvascular red blood cell flux (a function of red blood cell velocity and concentration) rather than blood volume [20]. During venous outflow obstruction, red-blood-cell concentration can increase due to pooling, while red-blood-cell velocity and local perfusion pressure decrease, which can yield a smaller and/or delayed net change in Flux [90,91,92]. However, because LDF is a single-point measurement with a small sampling volume [20], it is sensitive to spatial heterogeneity and probe replacement, which can reduce between-day reproducibility [22]. Additionally, delayed responses in PPG-derived PI suggest susceptibility to algorithmic smoothing or signal processing delays. These findings reinforce the need for more consistent and robust measures of tissue perfusion that are less vulnerable to motion artifacts, tissue heterogeneity, sensor placement variability, and delayed response. RTPI demonstrated sensitivity to both ischemic events in the analyzed datasets and exhibited immediate and significant declines at the onset of both occlusions. LDF has been reported to be less sensitive to compromised venous outflow and can exhibit a slower decline under venous occlusion than under arterial occlusion, which can make partial venous obstruction and congestion-like states harder to interpret than complete arterial stoppage [90,93]. Standard pulse oximetry and PI measures have also been shown to be unreliable for early venous congestion and may be inadequate indicators of venous occlusion [68,94]. Conversely, LDF is widely regarded as a sensitive method for detecting abrupt arterial occlusion [93], consistent with its slightly larger full-ischemia effect size in the present cohort.

Test–retest reliability of the protocol-contrast magnitudes was moderate for RTPI and poor for both Flux and PI, with wide confidence intervals that frequently crossed zero, indicating substantial between-visit variability in how strongly individuals expressed each contrast [65]. Therefore, none of the ICC values were strong. Because all three contrasts reflect local microvascular perfusion, their magnitudes are influenced by local vascular tone and autoregulatory control and are not determined solely by macrocirculatory hemodynamics once systemic pressure is within an adequate range [95,96]. Therefore, week-apart occlusion response magnitudes are physiologically expected to vary within the same participant, making only modest ICC (3,1) plausible even under an identical cuff schedule.

Because the ICC is essentially the ratio of between-participant variance to total variance, low or near-zero ICC values indicate that session-to-session variability dominates the ability to differentiate individuals by response magnitude consistently. The near-zero and negative ICC estimates observed for single-point LDF Flux are consistent with prior reports demonstrating high susceptibility of LDF to motion artifacts, spatial heterogeneity, and probe repositioning effects related to microvascular density inhomogeneity [22,97]. Moreover, because the evaluated contrasts represent regional cutaneous microcirculation rather than systemic flow, skin blood flow is reported to be more variable than muscle blood flow and to show large disparities from systemic variations, which makes week-apart repeatability of local response magnitudes inherently limited [98]. Similarly, low ICCs for PPG perfusion index are plausible given its dependence on intertwined central and peripheral factors, including vascular tone, stroke volume, thermoregulation, autonomic activity, and respiration, all of which can vary meaningfully between visits even under a fixed cuff protocol [46,47]. Negative ICC estimates can arise from sampling error when the true correlation is small, particularly in small samples, and are commonly treated as practical evidence of negligible reliability rather than true negative reliability [99].

Methodologically, these findings support the importance of physiological standardization, temperature control, and consistent probe placement for reference modalities in longitudinal studies, while also motivating multi-sensor or spatial-averaging strategies for LDF when long-term reliability is required [22].

RTPI’s multiparametric design and real-time responsiveness constitute a progressive advancement toward overcoming these limitations; however, further technical refinements and clinical validation are required.

The enhanced sensitivity of RTPI compared to traditional NIRS parameters was particularly noticeable during partial ischemia. RTPI responded immediately upon cuff inflation, whereas TOI initially increased before decreasing. This divergent response highlights the distinct physiological processes associated with venous outflow obstruction. Specifically, when venous drainage is impaired but arterial inflow persists, tissue oxygenation can transiently increase before subsequently declining as venous congestion progresses, consistent with prior venous-occlusion observations [93,100]. As venous congestion progresses, rising hydrostatic pressure within the microvasculature reduces the arteriovenous pressure gradient, restricting subsequent arterial inflow. Meanwhile, tissue metabolism continues to consume the trapped oxygen, thereby increasing HHb and delaying the decrease in TOI.

Similar hemodynamic mechanisms underlie the THb response. Under baseline conditions, the THb signal in a supine, healthy participant remains relatively stable, exhibiting only minor respiratory or vasomotor oscillations. When the cuff is inflated above venous but below arterial pressure, venous outflow is occluded while a reduced arterial inflow continues, leading to blood pooling and a progressive rise in THb. Each incoming pulse further distends the compliant venous reservoir, gradually increasing venous pressure toward the cuff level and diminishing the arteriovenous pressure gradient. As this gradient approaches zero, the rate of accumulation slows, and THb reaches a plateau, signaling the onset of functional ischemia. Although cuff inflation was limited to three minutes for participant comfort, it is plausible that a longer occlusion period would have revealed a subsequent decline in THb following the peak, analogous to the delayed TOI decrease observed during sustained venous congestion. The immediate drop in RTPI, in contrast to the simultaneous rise in both TOI and THb, demonstrates that perfusion-based parameters provide physiological information beyond conventional oxygenation metrics. This independence of NIRS-derived RTPI from tissue oxygenation values indicates its potential for real-time detection and management of tissue hypoperfusion in various clinical scenarios, including trauma, sepsis, and the entire postoperative continuum.

Absolute oxygenation indices derived from NIRS (e.g., StO_2_, rSO2, and TOI) are widely used as accessible surrogates of tissue perfusion or hypoperfusion in clinical practice and clinical research, particularly in shock and resuscitation settings. Decreased StO_2_ has been described as reflecting severe *hypoperfusion* and has been used clinically to guide resuscitation during hypovolemic shock [101]. StO_2_ has also been evaluated during trauma and ICU resuscitation as a surrogate for changes in oxygen delivery and as an adjunct for monitoring shock [102,103]. In septic shock, thenar StO_2_ and VOT-derived slopes are treated as *micro perfusion* parameters and studied alongside *macro perfusion* and metabolic variables [104]. In neonatology, tissue oxygenation is described as a surrogate for end-organ *perfusion* [105]. In this context, the phase-dependent dissociation we observe between RTPI and TOI/THb supports the value of extracting a perfusion-sensitive index from CW-NIRS to complement oxygenation-based surrogates, particularly for early transition detection in protocols that include venous outflow obstruction. This dissociation is physiologically expected because StO_2_ and TOI reflect the balance between regional oxygen delivery and consumption, and an apparently stable or even elevated oxygenation signal may arise from reduced oxygen extraction during hypoperfusion [106].

While this study successfully validated the NIRS-derived RTPI, several methodological considerations warrant discussion. First, although the sample size provided sufficient statistical power for initial validation, larger and more diverse cohorts are necessary to evaluate generalizability, establish normative thresholds, and assess inter-individual variability across different physiological and pathological states. Second, there were instrumentation-specific limitations affecting both references. LDF exhibited some sensitivity to probe placement, with signal quality in some cases improving only after minor repositioning. Consequently, analysis was restricted to three physiologically meaningful features previously supported in the literature, and feature selection pipelines (e.g., forward or backward selection, ablation tests) were not applied, as the reference itself could not be assumed to provide error-free ground truth for such procedures. Additionally, the cuff-release transition is abrupt and only partially controllable, such that the most significant feature-specific differences may be confined to brief transition windows. Future research will therefore incorporate protocols that include slower and more gradual perfusion-related dynamics than an abrupt cuff-release, such as orthostatic central hypovolemia challenges (e.g., head-up tilt) [107], graded simulated hypovolemia paradigms (e.g., multi-stage lower-body negative pressure) [108], and controlled respiratory challenges (e.g., voluntary breath-holding) [109], to better characterize feature kinetics over extended intervention periods.

Additional limitations should also be acknowledged. All measurements were conducted at a single anatomical site under resting, supine conditions with minimal autonomic variability; thus, RTPI performance under dynamic cardiovascular states warrants investigation. Moreover, because of unavoidable mismatches in sensor penetration depths, agreement between modalities in this study should be interpreted as consistency in the patterns of dynamic changes during controlled perturbations, rather than as evidence of matching absolute sampling depths or the same vascular compartments across devices. Furthermore, while test–retest reliability was assessed, the potential impact of sensor repositioning between sessions was not systematically evaluated. Additionally, although RTPI incorporates artifact-tolerant feature fusion, its robustness against motion remains to be tested under ambulatory conditions, which are critical for real-world wearable applications.

Moreover, because we did not apply active local fingertip heating in this study, and because perfusion in glabrous fingertip skin is strongly thermoregulated, even modest variations in ambient conditions or local skin temperature may have shifted baseline vasomotor tone and thereby altered finger perfusion signals, contributing to the observed variability [110]. Future studies could further reduce this confounder by continuously logging ambient temperature and, where feasible, applying standardized mild local warming using temperature-controlled finger microvascular assessment protocols to stabilize peripheral vascular tone during recordings [111].

A fixed 2-minute suprasystolic occlusion does not guarantee a uniform end-occlusion oxygenation minimum across individuals, because deoxygenation rates vary across participants and measurement configurations [39,40]. When oxygen depletion or oxygenation kinetics are primary outcomes, longer occlusions or target-based endpoints are preferable for stimulus standardization [38,42]. In the present study, conclusions are based on perfusion-sensitive indices and their repeatability/agreement under identical perturbations rather than on absolute end-occlusion oxygenation minima.

Lastly, spatial heterogeneity in perfusion across digits may have attenuated inter-system correlations, and treating finger measurement sites as interchangeable represents a methodological approximation rather than true physiological equivalence.

Several avenues for future investigation emerge from this work. First, the acceptable performance of RTPI in healthy participants supports its extension to clinical populations requiring tissue perfusion assessment and monitoring, such as individuals with peripheral arterial disease, diabetes-related microvascular complications, or patients undergoing reconstructive surgery where sustained flap viability monitoring is essential. In particular, the prefrontal cortex represents a promising target for cerebral applications: combining RTPI and TOI, both derived from one prefrontal NIRS sensor, may enable a multimodal approach for real-time monitoring of cortical perfusion and oxygen delivery during surgery, sedation, or critical care. Second, applying dynamic physiological stressors, including exercise, orthostatic maneuvers, and paced respiration, will facilitate assessment of RTPI stability under varying autonomic and cardiovascular conditions. Third, expanding the anatomical scope of RTPI measurement to include the extremities, surgical flaps, and internal organs using implantable NIRS sensors will enhance its clinical versatility [112]. Finally, implementing graded occlusion protocols with stepwise increases in pressure will enable high-resolution characterization of pressure–perfusion relationships and support the definition of physiologically and clinically relevant ischemic thresholds. This capability may open new avenues for the early diagnosis of acute compartment syndrome conditions, an urgent need that remains unmet in current clinical practice [113,114].

While the computational efficiency of PCA supports real-time RTPI computation, further optimization is needed to refine signal processing algorithms and ensure seamless integration into existing patient monitoring systems. Despite the intrinsic limitations of optical concentration-based techniques, specifically their inability to capture flow velocity, our findings demonstrate that unsupervised dimensionality reduction can extract clinically meaningful perfusion dynamics. Looking ahead, machine learning models trained on laser Doppler flowmetry data may enable the identification of NIRS-derived features that approximate flow velocity, potentially bridging the gap between concentration-based and velocity-sensitive perfusion assessments. However, such approaches must also address key limitations of LDF itself, including shallow tissue penetration and vulnerability to motion artifacts, to ensure robust, generalizable performance across clinical scenarios.

## Conclusion

This study introduced and validated the Regional Tissue Perfusion Index (RTPI), a novel NIRS-derived metric for continuous, non-invasive monitoring of regional tissue perfusion. By integrating multiple physiologically meaningful NIRS signal features through principal component analysis, RTPI achieved acceptable correlations with established reference standards while maintaining computational efficiency suitable for real-time applications. Importantly, RTPI demonstrated superior sensitivity compared with conventional NIRS-derived oxygenation indices, detecting immediate perfusion changes during both complete and partial ischemia, whereas oxygenation metrics exhibited delayed responses.

The multiparametric design of RTPI enables a more comprehensive characterization of microvascular dynamics than any single feature alone, establishing it as a robust and physiologically relevant alternative for bedside monitoring. These findings position RTPI as a promising tool for clinical applications across critical care, trauma medicine, perioperative and postoperative monitoring, vascular diagnostics, and rehabilitation.

Future work should aim to validate RTPI across diverse patient populations, extend measurements to multiple anatomical sites, and integrate the index into clinical monitoring platforms. Its ability to detect perfusion compromise independently of oxygenation indices highlights its potential value in addressing current diagnostic gaps, particularly in the early recognition of microvascular dysfunction. Finally, combining RTPI with advanced signal processing and machine learning techniques may further enhance its accuracy and scalability, paving the way toward reliable and clinically actionable monitoring of tissue health.

## Supplementary Information

Below is the link to the electronic supplementary material.

Supplementary Material 1

## Data Availability

No datasets were generated or analysed during the current study.
