# Supplementary material for: Regional tissue perfusion index (RTPI): a new optical-based metric for quantifying regional tissue perfusion
Source: J Clin Monit Comput. 2026 Feb 24;40(4):785–807. doi: 10.1007/s10877-026-01424-0 (PMC13391701; doi:10.1007/s10877-026-01424-0)
Supplement: Supplementary file 1 — Supplementary Material 1 [file 10877_2026_1424_MOESM1_ESM.docx]

**Supplementary Information**

This file reports additional analyses performed to address potential carryover between provocations and to provide full protocol-state discrimination statistics. These results are provided for completeness and transparency and are referenced in the main text.

**S.1 Carryover assessment between provocations**

Baseline-end and pre-partial values were not equivalent for any metric (Table S1). For Flux, Δcarryover was -26.42 with a 90% CI of [-39.54, -13.29] against an equivalence margin of ±7.26. For PI, Δcarryover was -1.52 (90% CI [-2.13, -0.92]) against ±0.19. For RTPI/PC1, Δcarryover was -1.20 (90% CI [-1.44, -0.97]) against ±0.18. Despite these baseline offsets, end-of-Recovery 1 trends were near zero on average (Table S2), suggesting the signals were largely stable, although they had not fully returned to baseline before the 100 mmHg occlusion. Because baseline-end and pre-partial values were not equivalent, partial-ischemia discrimination analysis was evaluated using both baseline and Recovery 1 as reference states.

**Table S1 - Baseline-end versus pre-partial equivalence two one-sided tests (TOST) results.**

| Metric | Δcarryover mean (90% CI) | Equivalence bound (±δ) | Equivalent? |
| --- | --- | --- | --- |
| Flux | -26.42 (-39.54, -13.29) | ±7.26 | No |
| PI | -1.52 (-2.13, -0.92) | ±0.19 | No |
| RTPI | -1.20 (-1.44, -0.97) | ±0.18 | No |

**Table S2 - Pre-partial window stability (Recovery 1 end slope).**

| Metric | Slope mean (95% CI), units/min |
| --- | --- |
| Flux | 0.11 (-5.91, 6.14) |
| PI | -0.02 (-0.25, 0.21) |
| RTPI | 0.02 (-0.10, 0.14) |

**S.2 Protocol-state discrimination statistics**

Protocol-state discrimination analysis (Table S3, Table S4, and Table S5) showed that RTPI decreased significantly during both occlusion phases relative to baseline and recovery when evaluated using protocol-defined phase labels rather than reference-modality correlations. During partial ischemia (100-mmHg), RTPI exhibited the largest standardized change (Cohen’s d_z_ = −5.02), exceeding the corresponding effects for Flux (d_z_ = −2.56) and PI (d_z_ = −1.78). During complete ischemia (200-mmHg), Flux showed a slightly larger standardized change (d_z_ = −4.18) than RTPI (d_z_ = −3.59), while PI showed smaller changes (d_z_ = −2.00). All planned contrasts remained significant after Holm correction (all p_Holm_ ≤ 3.32×10⁻⁶), indicating robust within-participant discrimination of protocol states.

**Table S3 - Discrimination of complete ischemia vs baseline**

| Perfusion metric | Mean paired change (Δ)* | SD of paired change | Standardized change, d_z_  (bootstrap 95% CI) | Holm-adjusted  p-value |
| --- | --- | --- | --- | --- |
| Flux | −168.45 | 40.26 | −4.18 (−6.06, −3.42) | 2.76×10⁻¹¹ |
| PI | −4.90 | 2.45 | −2.00 (−3.04, −1.53) | 1.11×10⁻⁶ |
| RTPI | −3.75 | 1.04 | −3.59 (−8.36, −2.53) | 1.01×10⁻¹⁰ |

*****Δ computed as (segment B − segment A); negative values indicate a decrease during occlusion relative to the comparator segment.

**Table S4 - Discrimination of partial ischemia vs baseline**

| Perfusion metric | Mean paired change (Δ)* | SD of paired change | Standardized change, d_z_ (bootstrap 95% CI) | Holm-adjusted  p-value |
| --- | --- | --- | --- | --- |
| Flux | −108.59 | 42.36 | −2.56 (−4.40, −1.75) | 2.53×10⁻⁸ |
| PI | −3.73 | 2.10 | −1.78 (−2.32, −1.51) | 3.32×10⁻⁶ |
| RTPI | −3.16 | 0.63 | −5.02 (−8.51, −3.84) | 1.70×10⁻¹² |

*****Δ computed as (segment B − segment A); negative values indicate a decrease during occlusion relative to the comparator segment.

**Table S5 - Discrimination of partial ischemia vs first recovery.**

| Perfusion metric | Mean paired change (Δ)* | SD of paired change | Standardized change, d_z_  (bootstrap 95% CI) | Holm-adjusted  p-value |
| --- | --- | --- | --- | --- |
| Flux | −87.96 | 37.57 | −2.34 (−3.99, −1.54) | 4.48×10⁻⁸ |
| PI | −2.72 | 1.53 | −1.78 (−2.52, −1.45) | 3.32×10⁻⁶ |
| RTPI | −2.43 | 0.65 | −3.74 (−6.06, −2.90) | 1.01×10⁻¹⁰ |

*****Δ computed as (segment B − segment A); negative values indicate a decrease during occlusion relative to the comparator segment.
